# Correlation of Doppler parameters with renal pathology: A study of 992 patients

**DOI:** 10.3892/etm.2013.1442

**Published:** 2013-12-10

**Authors:** QINKAI CHEN, FENG HE, XIAORAN FENG, ZHENGMAO LUO, JIANLIN ZHANG, LI ZHANG, YU WANG, JUNRONG TONG

**Affiliations:** 1 Department of Nephrology, First Affiliated Hospital of Nanchang University, Nanchang, Jiangxi 330006, P.R. China; 2 Department of Nephrology, First People’s Hospital of Jiujiang City, Jiujiang, Jiangxi 332000, P.R. China; 3 Department of Nephrology, General Hospital of Guangzhou Military Command of PLA, Guangzhou, Guangdong 510010, P.R. China

**Keywords:** Doppler, peak systolic velocity, end-diastolic velocity, resistive index, renal pathology

## Abstract

Ultrasound examination is a non-invasive diagnostic technique that is used on patients with suspected or established renal disease. The purpose of this study was to determine the role of intrarenal Doppler ultrasonography in the assessment of the renal pathology of patients with chronic kidney disease (CKD), as shown by kidney biopsy. This retrospective analysis enrolled 992 consecutive patients with CKD who underwent intrarenal Doppler ultrasonography and a kidney biopsy at the Departments of Nephrology of three hospitals between January 2006 and December 2010. Doppler parameters, including the peak systolic velocity (PSV), end-diastolic velocity and resistive index (RI) of the interlobar arteries, were compared with the renal biopsy findings. The RI of the interlobar arteries was correlated with the estimated glomerular filtration rate and the histological damage scores, demonstrating the most evident correlation with the tubulointerstitial damage (TI) score among the three histological components. The PSV of the interlobar arteries increased as the CKD stage progressed and correlated with a number of the renal histological changes, including the glomerulosclerosis and TI scores. The RI and PSV of the interlobar artery are correlated with the histopathological pattern in CKD. Thus, the RI and PSV of the interlobar artery may be potential indicators for monitoring the progression of renal damage.

## Introduction

Chronic kidney disease (CKD) is a risk factor for end-stage renal disease (ESRD). Early detection and timely management are essential for the medical care of patients with CKD. In clinical practice, information derived from kidney biopsies is commonly considered a *‘*gold’ standard that establishes the histopathological patterns concerning renal injury. However, biopsies are invasive and may result in various complications, including gross hematuria, and major complications that may eventually result in renal failure (1). Thus, certain disorders, including coagulopathy, are contraindicated for biopsy. Alternatively, Doppler sonography is a noninvasive method of examination that is widely used for the evaluation of patients with CKD.

Morphological changes associated with renal dysfunction, including the size, parenchymal echogenicity and corticomedullary differentiation of the injured kidneys, may be shown by sonography. However, these parameters lack specificity in the assessment of renal failure (2). Furthermore, it has been reported that the morphological changes detectable by sonography appear much later than the biochemical indicators, including increased serum creatinine levels (3).

An increased resistive index (RI) has been reported to correlate with glomerulosclerosis (GS), tubulointerstitial damage (TI) and vascular lesions (4). However, the results are not consistent (5). More notably, a previous study regarding renal histology has investigated small populations, and correlation analysis between reduced Doppler velocity in the interlobar arteries and histopathological changes in the impaired kidney is lacking (6).

The present study aimed to evaluate the correlations of a number of Doppler parameters, not only the RI but also the peak systolic velocity (PSV) and end-diastolic velocity (EDV), with histopathological changes in order to determine their significances in clinical decision-making concerning the care of patients with CKD.

## Materials and methods

### Patient information

A retrospective study was conducted and the medical records of 992 consecutive patients with CKD who underwent treatment between January 2006 and December 2010 at the Departments of Nephrology at the First Affiliated Hospital of Nanchang University (Nanchang, China), the General Hospital of Guangzhou Military Command of PLA (Guangzhou, China) and the First People’s Hospital of Jiujiang City (Jiujiang, China) were reviewed. The CKD diagnostic criteria used in this study fulfilled the guidelines proposed by the Kidney Disease Outcomes Quality Initiative of the National Kidney Foundation (7), and the classifications were made as follows: Stage 1, estimated glomerular filtration rate (eGFR, ml/min/1.73 m^2^) >90; stage 2, eGFR = 60–89; stage 3, eGFR = 30–59; stage 4, eGFR = 15–29; and stage 5, eGFR <15 or dialysis. For each patient, the age, gender, blood pressure, urinary protein, serum creatinine levels and eGFR at the renal biopsy were recorded. The study was conducted in accordance with the Declaration of Helsinki and approved by the Research Ethics Committee of the First Affiliated Hospital of Nanchang University, the General Hospital of Guangzhou Military Command of PLA and the First People’s Hospital of Jiujiang City. Prior to this study, informed consent was obtained from all patients.

### Doppler ultrasonography

Ultrasound evaluation was performed 24 h prior to the renal biopsy for all patients. In the maximum long-axis section images, the largest diameter and width of each kidney were measured. The patients were scanned in a supine or decubitus position to achieve an ultrasound beam as close to parallel to the blood flow direction in the intrarenal artery as possible. An ultrasound probe covered with transmission gel was gently placed on the skin over the kidneys. The PSV, EDV and RI were routinely measured as previously reported (8).

### Histological evaluation

The renal biopsy samples were evaluated for the severity of GS and TI damage based on previous scoring systems (9). Briefly, the GS score for each patient was evaluated in periodic acid-Schiff-stained sections and defined as follows: 0, normal GS; 1, matrix expansion or GS <25%; 2, GS = 26–50%; 3, GS = 51–75%; and 4, GS >75%. The TI score was assessed in azan- or periodic acid-methenamine silver-stained sections and defined as follows: 0, normal; 1, mild fibrosis around the vasculature; 2, mild fibrosis around the tubules; 3, moderate fibrosis with tubular casts or tubular damage; and 4, severe fibrosis with cell infiltration. The average score of the entire area of the biopsy sample of each patient was calculated for each histological component.

### Statistical analysis

All Doppler parameters were expressed as the mean ± standard deviation. SPSS software, version 16.0 (SPSS, Inc., Chicago, IL, USA) was used for statistical analysis. Student’s t-test was used to statistically analyze differences in the PSV, EDV and RI values in the main renal artery and interlobar artery between two groups. The correlations of the clinical and histological factors with the ultrasonographic indices were evaluated by stepwise multivariate regression analysis. P<0.05 was considered to indicate a statistically significant difference.

## Results

### Clinical characteristics of the patients

A total of 992 patients were diagnosed with CKD by renal biopsy, including 92 patients with IgA nephropathy, 109 with focal segmental GS, 103 with membranous nephropathy, 98 with minimal change disease, 149 with diabetic nephrosclerosis, 68 with crescentic glomerulonephritis, 32 with hypertensive nephrosclerosis, 134 with lupus nephritis, 114 with interstitial nephritis, 21 with amyloidosis, 17 with hereditary nephritis, 18 with hematopoietic stem cell transplantation-related nephropathy, nine with acute postinfectious glomerulonephritis and 28 with membranoproliferative glomerulonephritis.

The 992 patients underwent a renal biopsy and sonography. The baseline characteristics are shown in Table I. There were 112 patients with CKD stage 1, 278 with stage 2, 334 with stage 3, 198 with stage 4 and 70 with stage 5.

### Correlation of renal length with the CKD stage

**Figure 1:**
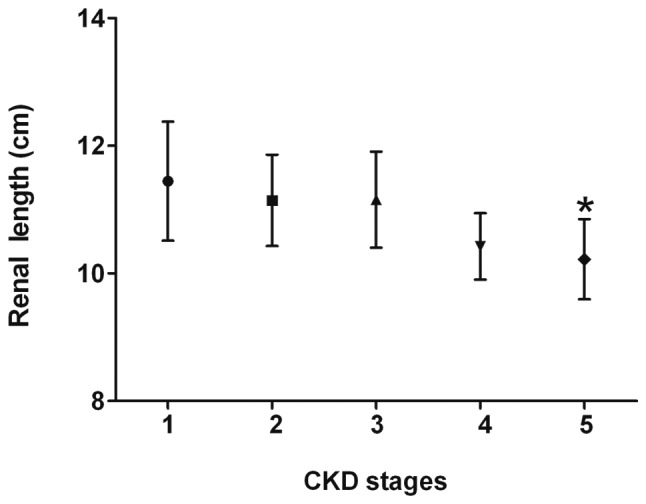
Changes in the kidney size based on the CKD stages. The renal length was significantly smaller in the patients with stage 5 CKD compared with that in the patients with stage 1, 2 or 3 CKD (P<0.01). However, in patients with stages 1–3 CKD, no clear association between the disease stage and kidney size was identified. ^*^P<0.05 versus stage 1, 2 or 3. CKD, chronic kidney disease.

The size of the kidneys was significantly reduced in the patients with stage 5 CKD, compared with that of the patients with stages 1, 2 or 3 CKD (Fig. 1). However, in the patients with stages 1–3 CKD, no significant association between the renal length and disease progression was identified, and the renal length showed a statistical but weak correlation with the renal function and histological damage scores (Table II).

### Correlation of RI with clinical and histological parameters

The RI was correlated with the renal function and histological damage scores and demonstrated the most evident correlation with the TI scores among the three histological components (Table II). However, it was not observed to be associated with the serum creatinine levels or CKD stage.

### Correlation of PSV and EDV with clinical and histological indices

**Figure 2:**
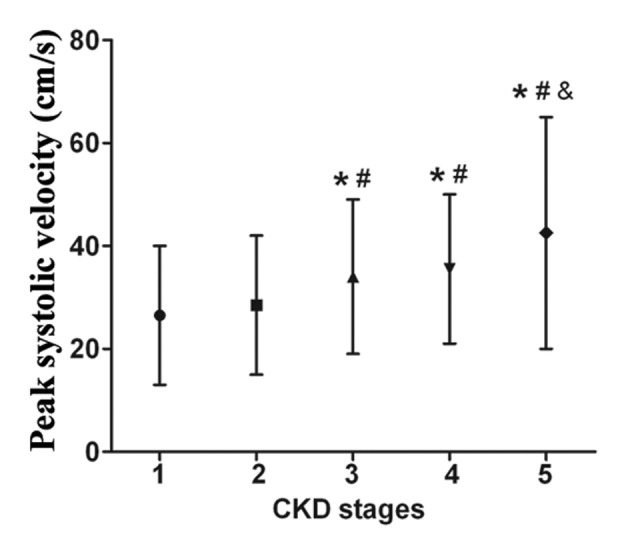
Changes in PSV based on the CKD stages. The PSV increased with CKD progression (P<0.01). ^*^P<0.01 versus stage 1, ^#^P<0.01 versus stage 2, ^&^P<0.01 versus stage 3. CKD, chronic kidney disease; PSV, peak systolic velocity.

No correlation of the EDV with the clinical and histological indices was identified. By contrast, the PSV increased as the CKD stage progressed (Fig. 2), and it was correlated with the GS (r=0.64, P<0.01) and TI (r=0.55, P<0.01) scores, showing the strongest correlation with the GS score among the three histological components (Table II). Stepwise multivariate regression analysis showed that the GS (β= −0.29, P<0.01) and TI (β=0.36, P<0.01) scores were risk factors for increased PSV in patients with CKD.

## Discussion

In the present study, the clinical significances of various parameters of Doppler ultrasonography, including the RI, PSV and EDV of the interlobar arteries, in patients with CKD were investigated. Among the ultrasonographic indices, the RI and PSV showed correlations with the renal function and histological damage scores. PSV was also a good marker of the CKD stage, but RI was not. By contrast, the renal length showed only a weak correlation with renal function.

Previous studies have shown that kidney length is often affected by the body size of a patient (10,11), and multiple conditions, including diabetic nephropathy and amyloid nephropathy, may also cause kidney enlargement. In agreement, the results of the present study showed that the parameter of kidney size was a poor indicator of the CKD stage.

In this study, the RI demonstrated a correlation with a number of histological parameters, and the most evident was observed with the TI score. Renal fibrosis, particularly tubulointerstitial fibrosis, is the common outcome of almost all cases of progressive or advanced CKD. Renal fibrosis is also a reliable predictor of prognosis and a major determinant of renal insufficiency (12). Consistent with the findings of the present study, a previous study has shown that renal arterial RI is associated with severe histological changes and poor renal outcome during CKD (13). Thus, RI may contribute to the identification of patients at a high risk of ESRD who may benefit from nephroprotective treatments.

One more important finding of the present study is that the PSV of the interlobar arteries increased as the CKD stage progressed and it correlated with renal histological changes, including the GS and TI scores. The PSV is a semiquantitative indicator of intrarenal blood flow on spectral Doppler imaging, and markedly depends on the distension of the small arteries in the kidney. Thus, the PSV is associated with renal vascular compliance and vascular resistance (14). The results of the present study suggest that PSV changes in the interlobar artery may be closely associated with a certain renal histopathologic type; however, the detailed mechanisms remain unknown.

Of note, the study does not suggest that any Doppler parameter in the intrarenal artery is able to replace renal biopsy. The present study sought to investigate the correlations between Doppler indices and certain renal histopathological parameters, including TI and GS. RI and PSV in the interlobar artery may potentially be used as indicators for detecting renal dysfunction. Further studies concerning Doppler parameters that have potential implications for the noninvasive evaluation of kidney damage are warranted.

## Figures and Tables

**Table I:** Baseline characteristics of the participants (n=992).

Variable	Value
Demographic	
Age (years; mean ± SD)	63±11
Male/female (n)	605/387
Body mass index (kg/m^2^)	24.41±3.52
Comorbidities	
Hypertension [n (%)]	641 (64.6)
Systolic BP (mmHg; mean ± SD)	134.6±33.7
Diastolic BP, (mmHg; mean ± SD)	84.7±23.2
Diabetes mellitus [n (%)]	293 (29.5)
Cardiovascular disease [n (%)]	281 (28.3)
Laboratory	
Hemoglobin (g/l; mean ± SD)	97.5±23.5
Serum creatinine (μmol/l; mean ± SD)	221.4±62.6
eGFR (ml/min/1.73 m^2^; mean ± SD)	21.2±5.9

BP, blood pressure; eGFR, estimated glomerular filtration rate.

**Table II:** Correlation coefficients (r) of the ultrasonographic parameters with the clinical and histological parameters (n=992).

Renal parameters	Renal length	RI	PSV	EDV
Serum creatinine levels	−0.11	0.10	0.32^a^	0.05
eGFR	0.29^a^	−0.31^a^	−0.03	−0.07
Glomerulosclerosis score	−0.27^a^	0.28^a^	0.64^a^	0.10
Tubulointerstitial damage score	−0.19^b^	0.43^a^	0.55^b^	0.09
CKD stage	−0.04	0.02	0.23^a^	0.06

a P<0.01,

b P<0.05.

RI, resistive index; PSV, peak systolic velocity; EDV, end-diastolic velocity; eGFR, estimated glomerular filtration rate; CKD, chronic kidney disease.
